# Why do we still have not a vaccine against Chagas disease?

**DOI:** 10.1590/0074-02760200314

**Published:** 2022-05-09

**Authors:** Erney Plessmann Camargo, Ricardo Tostes Gazzinelli, Carlos Médicis Morel, Alexander Roberto Precioso

**Affiliations:** 1 Universidade de São Paulo, São Paulo, SP, Brasil; 2 Fundação Oswaldo Cruz-Fiocruz, Rio de Janeiro, RJ, Brasil; 3 Fundação Oswaldo Cruz-Fiocruz, Centro de Desenvolvimento Tecnológico em Saúde, Rio de Janeiro, RJ, Brasil; 4 Instituto Butantan, Centro de Segurança Clínica e Gestão de Riscos, São Paulo, SP, Brasil

**Keywords:** Chagas disease control, prophylactic vaccines, therapeutic vaccines, vaccines production, vaccines evaluation

## Abstract

This review does not intend to convey detailed experimental or bibliographic data. Instead, it expresses the informal authors’ personal views on topics that range from basic research on antigens and experimental models for *Trypanosoma cruzi* infection to vaccine prospects and vaccine production. The review also includes general aspects of Chagas’ disease control and international and national policies on the subject. The authors contributed equally to the paper.

**AN OVERVIEW**

**Erney Plessmann Camargo**

Only four years after Carlos Chagas’s discoveries on *Trypanosoma cruzi*, Émile Brumpt attempted mice’s vaccination against the parasite.^1^ Following Jenner and Pasteur/Roux’s insights, Brumpt inoculated mice with non-lethal, attenuated strains of *T. cruzi* followed by a challenge with a virulent strain. Unfortunately, the inoculated mice exhibited only a “partielle immunité”, expressed by the reduced mortality but persistent parasitaemia. From there on, subsequent vaccination attempts by diverse methods and authors met with the curse of “Brumpt’s partial immunity”.

*Why was that, and why we still do not have a vaccine against Chagas disease? -* First, it was not for lack of interest and involvement of the scientific community worldwide, as Carlos Morel pointed out below. In fact, research on *T. cruzi* has always been on the Brazilian and international scientific agenda. In Brazil, in the 1970 decade, the Brazilian Research Council (CNPq), launched a wide-reaching Program of Research on Endemic Diseases (PIDE). Chagas disease was the program funds’ favored destination and attracted researchers from all research areas to work on *T. cruzi*, regardless of their objectives. As Professor Angelo Machado said at the time: “Hellas, now we can do basic research on an applied creature”. The PIDE also propitiated the Annual Meeting on Chagas disease to be held at Caxambu, MG, which facilitated the diffusion among scientists of Chagas disease’s social and political problems. An informal “*cruzi* community” was thus formed, which strongly influenced the adoption of the Control Program of Chagas disease in Brazil for the control of the domiciliary vector *Triatoma*, the “barbeiro*”* or kissing bug.^2^ ^,^ ^3^ ^,^ ^4^ The Southern Cone initiative, involving various Latin-American countries, soon followed the Brazilian control program.

The Caxambu meetings were equally crucial for disseminating modern molecular and immunological methods through the “trypanosome scientific community”. Among the research objects were tenths of vaccination assays, which used as antigens attenuated strains and cultural forms of *T. cruzi* killed by fixatives or inactivated by radiation or chemical agents blocking multiplication. Researchers also tried subcellular fractions, the most notorious of them being flagellar fractions, and even tried inoculation with non-pathogenic insect trypanosomatids. They moved to crude or purified antigens from cellular fractions, followed by specific molecular components of *T. cruzi* or ingeniously engineered molecules. The results of the modern biochemical and molecular attempts were, at the most, “encouraging”. Many attempts led to attenuated infections in which the mortality rates at the acute phase came to near zero or zero. However, when thoroughly investigated by xeno-diagnosis or by molecular methods, parasitaemias were seldom negative but always controversial.

In recent years, researchers began contemplating a serological and cellular response to antigens, thus clarifying many aspects of the CD8+ cells’ participation in response to *T. cruzi* infection. As discussed below by R. Gazzinelli, more recently, researchers began to try prophylactic and therapeutic DNA vaccines engineered on viruses.

Many of these experiments reported an attenuated acute phase followed by reduced or null mortality after challenge with a virulent strain. However, this is not equivalent to “cure”. As Zigman Brener and I pointed out in the eighties, “A vaccine which merely attenuates the acute phase of the infection - a procedure acceptable for some infectious diseases - would be of questionable value in Chagas disease”.^5^ In the disease’s pathogeny, organ damage is a chronic sequel of the acute and permanent infection, not an exclusive event of this phase. Hence, the absolute requirement for sterile immunity while searching for a prophylactic vaccine against Chagas disease. However, four decades ago, Brenner and I did not have the least idea about the eventual development and use of molecular, plasmidial, or viral engineered vaccines. Thus, our past emphasis on sterile immunity does not apply to therapeutic vaccines promoting a permanent attenuation of *T. cruzi* infections.

Concerning a fully protective vaccine against *T. cruzi*, many things conspired against its development, particularly the absence of a diagnostic method of cure and a better animal experimental model.

We have very efficient serological methods to detect past and present or acute and chronic infections used in nation-wide serological surveys.^6^ At the beginning of the 1980s, there appeared sensitive molecular methods for identifying strains of *T. cruzi* and circulating trypanosome DNA.^7^ The most successful vaccination attempts suppressed mortality at the acute phase, while the available methods confirmed the absence of residual parasitaemias. Unfortunately, these methods could not say much about the parasite’s eventual persistence in the intimacy of targeted tissues, as eventually revealed by exhaustive histopathological scrutiny.

Undoubtedly, most vaccination attempts yielded attenuated infections. However, attenuation does not equate with a cure, and we still do not have a sound diagnostic method for “cure” in Chagas disease, not only for asymptomatic human cases and for vaccinated animals but also for the testing of drugs.

I think that “mice” are part of the “diagnosis of cure” problem since mice are not the best model for testing vaccines against Chagas. All right, mice can be very useful as “first approaches” but are less useful for detecting chronic infections. Moreover, mice do not respond to some *T. cruzi* antigens recognised by human antibodies. Dogs seem to be a better model. In nature, dogs are infected by *T. cruzi* and are reliable epidemiological sentinels. They mirror well the human infection and develop a chronic pathology. However, researchers seldom use dogs in vaccination or drug assays.^8^ Psychological sensibilities and social pressure may render difficult the large-scale utilisation of dogs as an experimental model. Nevertheless, dogs could serve better than mice in the quest for a Chagas vaccine.

An effective vaccine against *T. cruzi* must also be effective against its seven known strains. Researchers have not thoroughly examined this possibility. Since Brumpt, we know about *T. cruzi* strains, but today we also know that they occur as distinct genotypes whose geographical distribution and natural reservoirs are known.^9^ Potential vaccines should be tested against the epidemiologically relevant genotypes infecting man and should prove effective against them all.

Also, a vaccine should not induce autoimmunity. In the 1940s, Muniz showed that chemically killed cultural forms of *T. cruzi* inoculated in *Rhesus* monkeys induced clinical and histopathological ‘hyperergic” myocarditis,^10^ but there is no contemporary evidence that *T. cruzi* infections cause autoimmunity in humans. Autoimmunity has been a hot issue in Chagas vaccines’ history, but it is no longer a leading cause of concern in vaccination assays.^11^

All our efforts to control Chagas disease centred on triatomine-mediated and blood transfusion transmission. These efforts produced such remarkable results to minimise the urgency for a prophylactic vaccine for Brazil and a few other endemic countries. These efforts did not intend and did not eradicate the agent or the vector and reservoirs of Chagas disease. Such an enterprise would be not only impossible but also ecologically undesirable. Thus, occasional, restricted episodes of Chagas infection remained. Unfortunately, recent oral transmission of *T. cruzi* in the Occidental Amazonian region is gaining increased epidemiological importance. Thus, a novel challenge and a new field are open to research and vaccination attempts considering the oral infection route.^12^

The comforting final word (if there is any comfort on that) is that we are not alone in the failure in search of a vaccine against a protozoan infection. Chagas disease, toxoplasmosis, amebiasis, and sleeping sickness also need a vaccine, while a recombinant vaccine against malaria still is under field testing. A vaccine lacks even for the African animal trypanosomiasis (AAT), for which experimental models are readily available. These facts suggest that protozoan vaccines may not follow the canonic models of bacterial and viral vaccines and require novel approaches.

Despite the shortcomings, we may rest confident that we will have an effective Chagas vaccine sooner or later. However, this will not be the last of our problems, and a new challenge will follow: how to test, manufacture, and produce a vaccine for humans, a costly process that may take years, as discussed by A. Precioso below.

**Science, markets, and health policies: facilitators or barriers to a Chagas disease vaccine?**

**Carlos Médicis Morel**

I was introduced to the fascinating field of Chagas disease and *T. cruzi* back in the 70s’, thanks to my colleague Isaac Roitman at the Cell Biology Dept (CEL) of the Institute of Biology of the University of Brasilia (UnB). It was Isaac who convinced me to attend the 1975 Caxambu meeting on Basic Research in Chagas Disease. Listening to an instigating talk by Zigman Brenner, urging participants to develop practical ways to characterise and differentiate the several “strains” of *T. cruzi* he kept at his laboratory, I was “hooked” by his challenge. I left Caxambu with a fresh idea in my mind: “Would the molecular biology techniques that I learned during my stay at the Institut Suisse de Recherches Expérimentales sur Le Cancer (ISREC) in Lausanne, Switzerland, and was adapting to our lab at the CEL/UnB, be of any help?” In collaboration with Isaac, Erney (then at the “Escola Paulista de Medicina” in São Paulo), Alvaro Romanha at the René Rachou Centre (Fiocruz), Belo Horizonte, and Larry Simpson at the University of California in Los Angeles, this idea soon turned into a reality.^7^ Characterising kinetoplastid pathogens became my major research area in the next 20 years and the driving force behind the Biochemistry and Molecular Biology Dept (DBBM) I created and directed at the Oswaldo Cruz Institute (IOC/Fiocruz), from 1978 until 1993, when I became Fiocruz President.

Having been trained as a molecular biologist and not as an immunologist - when these disciplines were still quite compartmentalised - I always kept a safe and respectful distance from the area of vaccines. I realised it was too complicated, too difficult, and too challenging. Thus, I decided it would be more realistic to work on “easier stuff” - molecular characterisation of *T. cruzi* and *Leishmania* strains, pushing for a parasite genome project and, perhaps, developing a molecular diagnostics approach for Chagas and other kinetoplastid diseases.

In 1998 I was nominated Director of the UNDP.World Bank/WHO Special Programme for Research and Training in Tropical Diseases (TDR). Leaving behind my former senior researcher positions, Director of the Oswaldo Cruz Institute (IOC/Fiocruz) and President of Fiocruz, to command a major international training and R&D program at the World Health Organization in Geneva was a new and demanding challenge. However, it also represented an incredible opportunity to meet new people and profit from interacting with some of the world’s best scientists and public health officials. One of them was Barry Bloom, from Harvard School of Public Health: trained in immunology, he has made important contributions to infectious diseases, vaccines, and global health policy. Barry is a brilliant person, a leader in immunology, and someone with significant political global influence, as he is often requested to speak at the US Congress on public health issues. During my stay in TDR, I met him several times, and we became close colleagues, as he used to thank me for getting approval from TDR’s Joint Coordination Board to include tuberculosis in the TDR disease portfolio in 1999. Among the issues I discussed with Barry, I remember very well two subjects, which I address here in his own words: “Carlos, have you an idea of how difficult it would be to get an effective vaccine against tuberculosis? All the years, we would have to wait to be able to measure the effect. All the investment it would need?” He was always pointing to the time and cost issues in the area of vaccines. He used to say, “if it is very difficult to develop a TB vaccine, a global disease, imagine the difficulties you would have with parasitic diseases that affect poor countries!”; Barry was very supportive but also quite critical of the TDR program. During one lunch we had, only the two of us, he said: “Carlos, they shaped TDR as if the problem of getting vaccines or drugs for tropical diseases would be only a *science failure*! It is not! You can publish as many papers as you would like, but the problem comes after that. Who would finance the development? Who would manufacture? Who would buy these new interventions? How to make it available in developing countries?”.

During my period as Director TDR, from July 1998 until December 2003, I saw the Human Genome Project’s (HGP) success, the creation of the Bill & Melinda Gates Foundation, and the sanitary crisis due to severe acute respiratory syndrome coronavirus (SARS-CoV-1). Even though the technological advances triggered by the HGP and the Gates to support a malaria vaccine (and recently to the idea of “malaria eradication”), progress in vaccines against parasitic diseases has been incremental but not transformative. These facts indicated that my generation (I was born during WW II) would not see the deployment of a viable, effective, and affordable Chagas disease vaccine.

The unmet promises of the HGP led to the concepts of “valleys of death” to the new area of work of “translational research” (or translation medicine, or translational science), bringing new life to the relevance of health innovation.^13^ Trying to address these issues, and remembering my conversations with Barry, I proposed, together with a few colleagues, that a global health innovation system (GHIS),^14^ would have to cope with science failures, market failures, and public health failures introducing the concept of “innovative developing countries” (IDCs).^15^ These concepts and frameworks are my minor contribution to a long-term perspective of the challenges ahead in vaccine development against parasitic diseases.

**Contemporary attempts towards a human vaccine**

**Ricardo Tostes Gazzinelli**

The advance in the fields of cell host-parasite interaction and immunological mechanisms of host resistance to infection associated with the omic projects, recombinant technology, and high throughput approaches led to enormous progress in vaccinology. The acquired knowledge and the use of these technologies allowed the discovery of novel candidate antigens and elaborated on effective vaccine formulations using the mouse model of Chagas disease, but little has happened to develop a human vaccine.

*Natural infection and disease* - *T. cruzi* has three stages that are relevant for the natural infection and perpetuation in the vertebrate host. The metacyclic trypomastigote, released by the triatomine vector, is the infective form of the parasite. This parasite stage stays in contact with the vertebrate host for only a few hours, and effective immunity against the metacyclic stage may lead to sterile immunity. The other two relevant stages are the intra-cellular amastigotes and extracellular trypomastigotes cycling in the host throughout the acute and chronic infection. Immunity to these parasite stages seems to keep the infection under control. The parasitaemia is submicroscopic in chronically infected individuals, and the parasites are only detectable by polymerase chain reaction (PCR), long term hemocultures, or xenodiagnosis. Re-infection with clinical signs of disease is rare, indicating that a robust immunity is induced by natural infection with *T. cruzi*. However, the infection never clears. Thus, a vaccine that targets the amastigotes and trypomastigotes may be beneficial by maintaining a low burden of parasites but is unlikely to promote sterile immunity.

*The immunological mechanisms of host resistance to infection* ^16^ - Immunity to the metacyclic trypomastigotes received little attention. Regardless, an effective vaccine to this parasite stage would block the parasite’s entrance into the host cell. Thus, an antibody-mediated immunity would be more efficient for killing or blocking the host cell’s parasite entrance. However, *T. cruzi* is a highly promiscuous parasite that can infect any nucleated host cell in addition to having diverse lineages. Thus, the parasite may use different surface antigens and different receptors to invade host cells. It is noteworthy that if only a few parasites escape the immune response, they are enough to invade the host cell, transform into amastigotes, and then into blood trypomastigotes leading to a productive infection.

Understanding immunological mechanisms of host resistance to amastigotes and trypomastigotes stages advanced a lot with the use of the genetically engineered mouse models of Chagas disease. Immune-mediated resistance to primary infection with *T. cruzi* is initially dependent on innate immune receptors, named Toll-like receptors that sense parasite DNA and RNA. These receptors are important because they initiate the production of IL-12 that triggers the production of IFN by NK cells and newly differentiated CD4^+^Th1 cells. However, parasite-specific antibodies are still low at this stage of infection, and CD8^+^ cytotoxic T cells are the central effector cells controlling parasite burden. The CD8^+^ T cells recognise antigens presented by MHC class I expressed by most nucleated cells and are vital for patrolling intracellular pathogens.

Like viruses, the *T. cruzi* amastigotes replicate in the host cell cytosol and are efficiently presented by MHC I and targeted by CD8^+^ T cells. The principal mechanism by which CD8^+^ T cells protect against *T. cruzi* infection is through IFN production. However, recent studies show that these CD8^+^ T cells contribute to *T. cruzi* infection’s immunity by destroying the infected host cells and the intracellular amastigotes.

For extracellular blood trypomastigotes, antibodies are the primary mechanism of protection. Evidence comes from vaccinated CD8^+^ T cell-deficient mice that become resistant to primary infection with *T. cruzi*. *In vitro*, antibodies directly kill the parasite both in a complement-dependent and -independent manner. Also, antibodies seem to mediate the protection through opsonisation and enhanced phagocytosis by macrophages. Antibody neutralisation and blocking of cell host invasion also play an important role in host resistance to *T. cruzi* infection.

*Discovery of antigen candidates for a vaccine* - The *T. cruzi* species diverge in various lineages differentially distributed in distinct geographical areas of Latin America. Hence, a major challenge to develop an efficacious vaccine against Chagas disease rests in the variability of surface antigens in different *T. cruzi* lineages. To have a universal vaccine for Chagas disease, one would need to identify conserved antigens in the multiple parasite lineages. Conserved antigens, shared by metacyclics, amastigotes, and blood trypomastigotes stages of the parasite, would be ideal targets for an effective Chagas disease vaccine, but such shared antigens received little attention. However, immunoproteomics, immunopetidomics, and Chagas disease’s mouse model revealed many vaccine candidates for a Chagas’ vaccine. Among them, we can include a member of the active trans-sialidase (aTS) family, the amastigote surface antigen 2 (ASP-2), a big TS family member Tc24, ribosomal proteins, and cruzipain.^17^ ^,^ ^18^ ^,^ ^19^ The most studied vaccine candidate antigen of *T. cruzi* is the aTS.

The aTS start to be expressed by the blood trypomastigotes just before the parasites leave the host cells. Protective antibodies against *T. cruzi* infection in mice recognise both the active and the shed acute phase antigen (SAPA) domains composed of tandem epitope repeats. Also, the TS and ASP2 epitopes that are recognised by CD8^+^ T cells have been identified. Amastigotes express ASP2 but induce a relatively low antibody response. The association of ASP2 and TS as a vaccine induces antibodies that target the extracellular blood trypomastigotes and the CD8^+^ T cell-mediated immunity (TCMI) that target the intracellular amastigotes. This antigen combination induces a highly effective protective immunity against experimental infection with *T. cruzi* in mice, often leading to parasite clearance from the host.

Another important vaccine-candidate for Chagas disease is the αGal epitope, a terminal sugar present in different glycoproteins expressed on the surface of different lineages of *T. cruzi*. While this epitope is the main target of antibodies present in sera of patients with acute and chronic Chagas disease, it has not been studied in mice since they express high levels of αGal and do not produce antibodies to the terminal αGal. Interestingly, recent studies used the α1,3-galactosyltransferase knockout mouse that lacks the αGal in host proteins. These mice produce elevated levels of anti-αGal antibodies, are more resistant to primary infection, and highly resistant when immunised with a protein carrier or viral-like particles (VLPs) particles covalently linked to the αGal epitope.

*Vaccine formulations* - While aTS, ASP-2, and αGal epitope are excellent vaccine candidates for Chagas disease, the context in which an antigen is presented is critical to elicit the desirable type of protective immunity. The quality of protective immunity varies from pathogen to pathogen. In the case of *T. cruzi* infection, an ideal vaccine should elicit CD4^+^ Th1 lymphocytes that produce IFN, which provide help for the production of protective antibody isotypes (IgG1 and IgG3 in humans) and activation of CD8^+^ cytotoxic T cells. Besides, the induction of immunological memory and long-term immunity is highly desirable to avoid multiple vaccine doses.

*Recombinant antigens* - Currently, the most explored vaccine formulations involve the use of *T. cruzi* recombinant parasite antigens produced in bacteria associated with immunological adjuvants that elicited T cell-mediated immunity. Vaccination with different *T. cruzi* recombinant antigens associated with specific adjuvants, while not sterile, induces strong protection for a few months after the last vaccine boost. They are highly effective in inducing CD4^+^ T cell response and high antibody levels but not effectively inducing CD8^+^ T cell response. Also, the induction of immunological memory and long-term immunity requires multiple doses of the recombinant antigens associated with adjuvants. Another limitation is the choice of the adequate immunological adjuvant. While there are many adjuvants used in mice, adjuvants’ availability to be used in humans is limited. On the other hand, a significant advantage of recombinant proteins is that the technology for scaling up and production in good-manufacturing practices (GMP) is well-established and cheaper than other alternatives.

*Plasmidial DNA* - An alternative formulation of simpler production that bypasses adjuvant use is the plasmidial DNA encoding parasite genes.^20^ The plasmidial DNA produced in bacteria is accessible to scale-up and to produce on a large scale. The plasmidial DNA is transfected into host cells, is expressed in the host cell cytoplasm, and therefore presented by MHC I to cytotoxic CD8^+^ T cells. However, they are very poor inducers of CD4 T cells and antibody responses. In the experimental model of Chagas disease, DNA-based vaccine protocols induce limited immune response and protection against the *T. cruzi* challenge.

*Viral vectors* - The use of attenuated viral vectors is a useful alternative for developing vaccines to elicit CD8^+^ T cell response and long-lasting immunity. Various attenuated viral vectors have been tested in experimental *T. cruzi* infection in mice. Among them, we include adenovirus (Ad), influenza, and the attenuated yellow fever vaccine virus encoding either an entire or a fragment of the aTS or ASP-2. The vaccination with attenuated viral vectors often requires a heterologous prime and boost protocol (HetPB). The HetPB protocol uses one dose with a specific viral vector and a second dose with either an alternative viral vector, plasmidial DNA, or even a recombinant protein. This procedure is relevant because the virus often elicits an immune response to its antigens and be cleared by the host immune response in the vaccination boost. In our studies, while not protective on their own, both influenza and the plasmidial DNA could enhance the protective effect elicited by the Ad-ASP-2 and Ad-TS. The vaccination using a priming dose with plasmids encoding both ASP-2 and aTS and the boosting dose with Ad-ASP-2 and Ad-TS is highly protective. Besides, in some studies, we have used the homologous prime-boost protocol (HoPB) to effectively vaccination against *T. cruzi*.

On the one hand, we found that HoPB protocol using Ad-ASP-2 and Ad-TS, simultaneously, or Ad-APS2 alone induced long-term immunity and was highly effective against *T. cruzi* challenge leading to sterile protection in approximately 50% of the mice. On the other hand, we found that the HoPB protocol using yellow fever attenuated vector-expressing fragments of ASP-2 induced only partial protection. Hence, in our hands, the adenovirus was the more effective platform, its main advantages being the cost of scaling up and industrial production. We also used the Ad-5, a human pathogen that infects a large fraction of the human population, producing high antibody levels. However, vaccination with Ad-5 maybe not effective in humans. One alternative is to use adenovirus from other species as vaccine vectors, which have been shown effective in different studies.

*Genetically manipulated attenuated parasites* - Experimental results revealed that highly attenuated parasites efficiently induce long-term protective immunity against *T. cruzi* parasites, even after a single vaccination dose. With the development of the CRISPR/Cas9 as a technology for gene manipulation in eukaryotic pathogens, new hope emerges for the generation of attenuated parasite strains as a vaccine for Chagas disease. The previously developed techniques for gene manipulation were not so effective against *T. cruzi* parasites. For instance, the homologous recombination was not effective because tens, hundreds, and even thousands of genes encode many of the *T. cruzi* vaccine candidates. Besides, RNA interference is not useful because the RNAi machinery is not present in *T. cruzi*.

In contrast, the CRISPR/CAS9 is highly efficient in deleting multiple gene copies of the parasite. The advantage of using the genetically manipulated parasites, rather than the naturally attenuated parasites, is that reversion is unlikely to happen, and therefore the vaccine is safe. The key to generating the attenuated parasite through CRISPR/CAS9 is the identification gene that is key for the rapid parasite proliferation *in vivo*, but not for parasite survival. We believe that in a live attenuated vaccine, the parasite presents limited proliferation and does not cause disease, but at the same time, survives for various generations to elicit long-term immunity. Our group has now generated substantial evidence that parasite attenuated with CRISPR/Cas9 technology induce very strong protection against experimental challenge with *T. cruzi* and do not cause disease in the immunodeficient host.

*The therapeutic vaccine* - Today, there are 5 to 8 million patients chronically infected with *T. cruzi.* Drug treatment has limited efficacy in these patients. Since domiciliary transmission was eliminated in many Latin American countries, having a therapeutic vaccine for Chagas disease is more relevant.^21^ There are at least three rationales to elaborate vaccine protocols to treat chronic disease. The first one is to build an effective immune response, impaired in chronically infected individuals. The second one is a combined therapy to enhance drug efficacy, and the third one would be the vaccination of patients after chemotherapy to prevent relapses and parasite proliferation in the cardiac tissue. Many of the aspects discussed above regarding the immunological mechanisms of protection and vaccine formulations also apply to developing a therapeutic vaccine for Chagas disease. Although the ideal vaccine formulation to treat patients may be different from the one used for prophylaxis. In planning the formulation of a therapeutic vaccine, one needs to anticipate that chronic Chagas disease patients already have an immune response to the parasite. It is conceivable that the disease is a result of an uncontrolled inflammatory response elicited by the parasite. In this case, a therapeutic vaccine that enhances the anti-parasite response may intensify tissue inflammation and aggravate the cardiac disease.

Not many studies have evaluated therapeutic vaccines in a mouse model of chronic Chagas disease. One of the main problems is that chronic disease in mice does not reflect human patients’ complex chronic disease. Dogs with chronic *T. cruzi* infections develop a cardiac disease similar to humans and maybe a better model. Nevertheless, few studies have used the formulations tested in prophylactic vaccines, which contained recombinant *T. cruzi* proteins or adenovirus expressing ASP-2 and aTS. These therapeutic protocols decreased parasitism and heart inflammation, suggesting their usefulness to limit parasite proliferation in infected individuals. Additional studies are required to evaluate the effect of combined therapy using vaccination and drug treatment.

*The delay in the development of an infective vaccine for human disease* - Despite the favorable results of experimental infections with *T. cruzi*, developing a human vaccine made little progress. Different reasons may explain this delay. In the first place, the concept that only a vaccine that elicits sterile immunity should apply to the Chagas vaccine. This is an extremely difficult goal to be achieved, if achievable, at all. Of note, some of the highly effective viral vaccines that are known to induce protection for life and believed to induce sterile immunity, such as yellow fever and measles, seem to induce a robust and long-term immunity due to the persistence of the attenuated viruses.

Other aspects are the limitation of testing a prophylactic vaccine in humans. Since the infection leads to chronic infection and its effectiveness is limited, its use of experimental challenge in humans is considered unethical. Furthermore, the debatable concept that cardiac Chagas disease is due to an autoimmune disorder elicited by *T. cruzi*-infection also limits attenuated parasites’ potential use as a vaccine against *T. cruzi* infection. Moreover, the long period that Chagas disease’s symptomatic forms take to develop makes it difficult to evaluate a prophylactic vaccine’s effectiveness for developing chronic disease. Also, considering that the transmission is very limited, a phase 3 clinical trial is difficult to plan. Fortunately, most of these logistic and ethical issues do not apply to a therapeutic vaccine’s clinical trials in chronic patients.

Finally, Chagas disease primarily affects the less privileged segment of our society, and, currently, the transmission is limited to a few geographic areas. Hence, the effort and costs of developing this vaccine maybe not of economic interest to the pharmaceutical industry. Therefore, government investment is necessary for scaling up GMP production and phase 2/3 clinical trials of a vaccine for Chagas disease.

**Development plan of a Chagas vaccine**

**Alexander Roberto Precioso**

A new vaccine’s manufacturing process encompasses distinct and specifics steps that make it a long-term, high-cost, and high-risk investment. Overall, the R&D for a new vaccine comprises four steps: basic research (the prove of concept phase), preclinical, and clinical phases (phases I to III). The pharmacovigilance (phase IV), which follows the licensure and has no predetermined duration.

The step of basic research belongs to the realm of science and has no practical or commercial commitments. Basic research on vaccines is carried out in laboratories *in vitro* and experimental animals *in vivo*. There are no specific legislation or guidelines for this phase, except for those dealing with animal misuse ethics. However, the resulting data has to be registered and analysed as “a prove of concept” for the vaccine under development. After that, the results are included in the Clinical Development Dossié to submit to ethical and regulatory approval.

Unlike the “research” phase, clinical studies must comply with Good Laboratory Practices according to vaccine testing and evaluation rules.

The clinical evaluation of a new vaccine follows the typical phase series from early, small-scale (phase I), late-stage (phase II), and large-scale trials (phase III). Before testing a new vaccine in humans, regulatory and ethical approvals are necessary, and they may vary among countries.

Phase I corresponds to the first time that a new vaccine is tested in humans. Therefore, it is done in a small number of volunteers, male and female young adults (20-100). The main objectives of phase I are to verify the new vaccine’s safety, tolerance, and reactogenicity. It also may serve to obtain immunogenicity data as well.

Phase II aims to demonstrate the immunogenicity and to increase the data on vaccine safety. This phase involves several hundred to a thousand volunteers of different age groups and diverse health/disease conditions.

The phase III studies are large-scale clinical trials designed to demonstrate the efficacy and provide an expanded safety assessment of the new vaccine. These studies are performed in large populations (thousands of volunteers) and are the last clinical evaluation stage before a new vaccine can be licensed.

Unlike the basic research environment, the vaccine’s manufacturing and clinical trials occur in a very regulated scenario of good manufacturing and clinical practices. Overall, a vaccine has to follow since the beginning the principles of the “target product profile” (TPP), which outlines the desired profile of the proposed vaccine.

Phase III of a new vaccine is the most time-demanding, most expensive, and most risky stage. Besides a very robust TPP, the clinical protocol must be designed by a very qualified team supported by specialists and conducted according to the good clinical practices before licensure. Overall, the main challenges associated with clinical trials are: (a) volunteer recruitment according to inclusion and exclusion criteria (recruitment of children and pregnant women are problematic); (b) definition of the target population for phase III; (d) assuring the participants’ retention and follow-up in phase III (the longer the follow-up period, the higher is the participants’ drop-out); (e) the definition of “disease case” and protection (against disease or infection), based on clinical data and specific and sensitive laboratory assays.

Bringing together all the issues described into the rationale for developing a Chagas vaccine, one faces specific questions such as a preventive or therapeutic vaccine to decrease the parasite burden, cardiac tissue inflammation, and damage, and increase survival. Has vaccination the potential to exacerbate disease by stimulating autoimmunity? Also, similarly to what happens in dengue, can the vaccine increase susceptibility to infection by inducing weakly heterologous neutralising cross-reactive antibodies and favoring a more severe secondary infection by a heterologous serotype of the pathogen. What is the feasibility of a Chagas vaccine clinical trial as the disease takes years to develop? Given the low incidence of the disease, would an efficacy clinical trial need a very large number of participants in phase III? What would be a feasible vaccine clinical trial since the disease takes years to develop?^22^ ^,^ ^23^ ^,^ ^24^

Finally, all the information generated in the development and manufacturing stage, combined with the preclinical and clinical steps, should be gathered and filed to get regulatory approval. After the regulatory approval, we can produce the vaccine and make it available to the people.

**Authors’ concluding remarks**

The simplification and increased reliability of the serological diagnostic tests led to a correct appraisal of the dimension of the Chagas’ endemics in Brazil in the 1970s. This memorable achievement underscored the launching of the Program for the Control of Chagas Disease. This program was soon followed by the South Cone initiative, involving many countries committed to eradicating the domiciliary vector of *T. cruzi.* Serological tests implemented at the blood banks also interrupted Chagas disease transmission by blood transfusion. The absolute success of the domiciliary vector control program minimised the urgency of a prophylactic vaccine.

Nevertheless, a prophylactic vaccine remains the gold standard for Chagas disease prevention in endemic countries, particularly Latin American countries, not benefited by the anti-*Triatoma* control programs. Thus, research efforts must continue in the search for a prophylactic vaccine. Simultaneously, vaccination strategies should contemplate the new epidemiological scenario in Brazil where focal upsurges of trypanosomiasis of oral transmission are regularly occurring in the Amazonian Region.

Alongside a prophylactic vaccine, considering the low efficacy of drug treatment for chronically infected individuals, a therapeutic vaccine must be an essential part of future research efforts. A therapeutic vaccine will be of utmost interest for countries plagued by Chagas disease and countries with sporadic autochthonous infections and increasingly receiving Latin American immigrants.

However, all these efforts will be frustrated whether we do not develop a reliable diagnostic test for the infection cure. Not only for testing a prophylactic or therapeutic vaccine but also for testing any eventual drug for the treatment of Chagas disease.

After the regulatory approval of a vaccine against *T. cruzi*, a problem remains: who will respond to the vaccine production’s high costs since it may not be of economic interest to the pharmaceutical industry.

However, the answer to this question transcends the scientific realm.
